# Dental health between self-perception, clinical evaluation and body image dissatisfaction – a cross-sectional study in mixed dentition pre-pubertal children

**DOI:** 10.1186/s12903-018-0542-2

**Published:** 2018-05-03

**Authors:** Ancuta Banu, Costela Șerban, Marius Pricop, Horatiu Urechescu, Brigitha Vlaicu

**Affiliations:** 1 Department 2, Discipline of Maxillofacial Surgery, Faculty of Dentistry, “Victor Babes” University of Medicine and Pharmacy Timișoara, 5 Take Ionescu Bvd, 300062 Timisoara, Romania; 2 Department 3 Functional Sciences, “Victor Babes” University of Medicine and Pharmacy Timișoara, 14 Spl. Tudor Vladimirescu, 300172 Timişoara, Romania; 3 Department 14 Microbiology, “Victor Babes” University of Medicine and Pharmacy Timișoara, 16 Victor Babes Bvd, 300226 Timișoara, Romania

**Keywords:** Self-perception of oral health, Mixed dentition, Decayed teeth, Body dissatisfaction

## Abstract

**Background:**

Self-perception of oral health status is a multidimensional construct that includes psychological, psychosocial and functional aspects of oral health. Contemporary concepts suggest that the evaluation of health needs should focus on clinical standards and socio-dental indicators that measure the impact of health/disease on the individual quality of life. Oral health cannot be dissociated from general health. This study evaluates a possible association between oral health status, body size, self-perception of oral health, self-perception of body size and dissatisfaction with body image in prepubertal children with mixed dentition, targeting the completion of children’s health status assessment which will further allow the identification of individuals at risk and could be further used as an evaluation of the need for specific interventions.

**Methods:**

The present study is cross-sectional in design and uses data from 710 pre-pubertal children with mixed dentition. The outcome variables comprised one item self-perception of oral health: dmft/DMFT Index and Dental Aesthetic Index, body size, self-assessed body size and desired body size. Multiple logistic regression analyses were performed. The level of significance was set at 5%.

**Results:**

More than a half (53.1%) of the participants with mixed dentition reported that their oral health was excellent or very good. In the unadjusted model, untreated decayed teeth, dmft score and body dissatisfaction levels had a significant contribution to poor self-perception of oral health, but after adjustment for gender, BMI status, dmft score, DMFT score and DAI score, only untreated decayed teeth OR = 1.293, 95%CI (1.120–1.492) and higher body dissatisfaction levels had a significant contribution.

**Conclusion:**

It was concluded that the need for dental treatment influenced self-perception of oral health in prepubertal children with mixed dentition, especially with relation to untreated decayed teeth. Since only body dissatisfaction levels, but not BMI, were related to poor self-perception of oral health, which involves a psychological component, further studies should evaluate the risk factors of body dissatisfaction, in order to plan health care directed to this age group, and with the purpose to positive parenting strategies.

## Background

Self-perception of oral health status is a multidimensional construct that includes oral disease, tissue damage, modified functional capacity, pain, and aesthetics - psychological and psychosocial components. Self-reported oral health status is a relatively simple and easy method of assessment, which can be routinely collected and has several uses, such as the assessment of perceived treatment needs, and as a monitoring tool for health promotion intervention. Although most studies on contributors to oral health perception targeted adult populations, there have been some studies performed on children and adolescents which have shown good consistency over time of one item self-rated oral health, and its importance in taking preventive measures [1, 2]. It has been concluded that self-reported oral health has an impact on both well-being and quality of life [3]. In children and adolescents, oral health-related quality of life has been proved to be impacted by several oral conditions such as dental caries or malocclusion, but also by psychological components such as self-esteem and positive self-image [4–8].

With the rise in prevalence of childhood obesity, several studies have attempted to explore the links between dental decay and obesity, concluding that they share common etiological, social and behavioural factors [9].

The association of weight status with the body image is formed through the subjective judgment of body size by children and has been demonstrated in numerous studies that have focused on satisfaction with body shape. Dissatisfaction with body image was judged to increase with age among the groups of children and adolescents studied [10]. A negative body image was correlated with depression, anxiety, low self-esteem, obsessive-compulsive behaviours [11], and inadaptive behaviours in adolescence [12].

### Objectives

Since it is demonstrated that effective prevention strategies improve the oral health status and considering the before-demonstrated link between the oral health perception and the previously mentioned items (body image, self-esteem and self-image) we might hypothesize that by identifying a cluster of target patients in which screening and pro-active prevention strategies will have an increased impact leading to an increased quality of life with all the associated benefits. Thus, to be able to improve the screening strategies and identification of most vulnerable patients, this study aimed to evaluate a possible association between oral health status, body size, self-perception of oral health, self-perception of body size and dissatisfaction with body image in prepubertal children with mixed dentition.

## Method

### Sample

Data was taken from the program for monitoring and control of oral health status of the population of Timișoara, Romania undertaken in the Maxillo-Facial Surgery Clinic of City Hospital Timișoara with the support of the City Hall in Timișoara and the Timiș County School Inspectorate. The program was promoted in all 40 Primary and Middle Schools from Timisoara, Romania, and all attending students were invited to participate. In Romania the school is compulsory until the age of 16, representing 11 grades: from the preparatory school year to the tenth grade. The sample was obtained by availability.

**The sample size** was considered as being representative of the city of Timișoara, which, at the last referendum had 319,279 inhabitants with a proportion of children attending school with age range 7–18 years of 9.4% [13]. Taking into account our actual sample size of 710 participants, the calculated margin of error for the sample is 3.6%.

Selection criteria for participants:

- inclusion criteria: children in the selected age group (8–11 years), with mixed dentition, of normal weight and overweight by WHO (World Health Organization) 2007 criteria, children without a serious general illness, cooperative children, children whose parents have given informed consent.
- exclusion criteria: underweight children (as defined by WHO 2007 criteria), children with exclusive temporary or definitive dentition, children with genetic or post-traumatic malformations, children with a serious general illness, children with rare conditions.

Out of the total of 945 children examined, a sample of 710 primary school children was obtained which had no missing data on outcome variables. The group had a mean age of 114 (+/− 11.3) months. 52.8% (375) of the sample were boys.

**Data collection** started in May 2015 and continued for 8 weeks. The children were evaluated clinically and with a questionnaire-based interview, performed on the same day. The children responded voluntarily to the personalized interview, which was adapted to their age, with questions covering the self-assessment of their oral health status, their perception of body image and their desired body shape. The following information was recorded: gender, date of birth, date of consultation. Evaluation was conducted by examiners licensed both in medicine and dentistry. The dental clinical examiner is maxillo-facial surgery specialist and has competence in Pedodontics and Orthopedic dentistry.

Weight was measured by a single examiner, in minimal clothing, without shoes, to the nearest 0.1 kg, with a calibrated mechanical step scale; height was measured to the nearest 0.5 cm under the same conditions using a standard stadiometer from the medical office.

**Body mass index (BMI)** was calculated from weight and height measurements, based on the following formula: BMI = weight (kg)/height (m)^2^. WHO AnthroPlus macros were used for assessing growth and adiposity status (BMI-z score). Furthermore, children were classified according to WHO recommendations of normal weight category, overweight and obesity [14].

**Perceived body image** and **desired body image** were measured using the Pictorial Body Image Instrument [15], a well-established method for assessing body image dissatisfaction, which facilitated the children’s choices and their communication with the examiner. The boys and girls were presented with a set of seven drawings of children, matched to the respondent’s gender, ranging in size from very thin to obese, and numbered from one (very thin) to seven (obese).

Body image dissatisfaction was calculated as the discrepancy between the perceived and the desired body images assessed as part of the questionnaire. A score of zero indicated that the child was satisfied with his/her size, a positive score indicated that the child wanted to be thinner, and a negative score indicated that the child wanted to be larger.

**The endo-oral clinical examination** was performed by a single examiner, in a dental chair, with the help of a mouth mirror, probe, and William’s probe which was used to determine the overjet and overbite. The following were recorded:

- number of teeth that are decayed (d for primary teeth, D for permanent teeth), missing (m for primary teeth, M for permanent teeth), or filled (f for primary teeth, F for permanent teeth) and were computed the dmft/DMFT index for mixed dentition, according to WHO recommendations [16]. dmft/DMFT index is established as the measure of caries experience in dental epidemiology.
- parameters Dental Aesthetic Index (DAI): overjet, underjet, missing teeth, diastema, anterior open bite, anterior crowding, anterior spacing, largest anterior irregularity (mandible and maxilla), anterior-posterior molar relationship.

The Dental Aesthetic Index were computed according to WHO recommendations [16]. It has two components: a clinical component and an aesthetic component and it links the clinical and aesthetic components mathematically to produce a single score that combines the physical and the esthetic aspects of occlusion. DAI was adopted as a cross-cultural index by the World Health Organization for the assessment of orthodontic treatment needs.

**Perceived oral health status** was measured on a 7 item Likert scale that comprised the following possible answers: excellent, very good, good, average, poor, very poor and I do not know option. The question was: “How would you rate your overall oral health?”. Since self-perceived oral health status is a latent construct, the best approach in registering the participants’ subjective opinion on this topic is a Likert-type ordinal scale answer.

### Data analysis

Data analysis was performed using the Statistical Program for Social Sciences for Windows (IBM-SPSS, Version 18). Perceived oral health status was the main outcome of the study. The main explanatory variables were clinical oral status (dmft score, DMFT score, number of untreated decayed teeth), BMI categories and body image dissatisfaction. Other variables included in the analysis were the participant’s gender and age. For numerical variables, descriptive summary measures of central tendency were computed. For ordinal or nominal variables, frequency (%/N) were computed. For comparisons between genders of continuous variables under the presumption of normality t-tests were used and for continuous variables which failed in the assumption of normality and homogeneity of variance, and categorical variables Man-Whitney test was used.

Using the clinical indicators (dmft score, DMFT score, DAI score and number of untreated decayed teeth) as dependent variables, four general linear models were used to test differences between gender (M/F) and age groups (8–9 years / 10–11 years) and their interaction, using Estimated Marginal Means, and comparing their main effects using Sidak correction for multiple comparisons. Using body dissatisfaction as a dependent variable, and gender, BMI categories and their interaction as predictors, another general linear model was constructed, applying Sidak correction for multiple comparisons.

Logistic regression was applied for the prediction of poor self-perception of oral health, which, after recoding self-perception of oral health, resulted in: excellent and very good as high self-perception of oral health and the rest of answers as poor self-perception of oral health. As independent predictors we have used gender, clinical oral indicators (dmft score, DMFT score, DAI score, total number of untreated decayed teeth), BMI categories and body dissatisfaction levels in absolute discrepancy. Univariate OR were computed separately for all independent variables. In the following step a model was constructed.

All statistical tests performed were 2-tailed and statistical significance was defined by a *p* value < 0.05.

## Results

### Self-perception of oral health

Descriptive statistics of self-perception of oral health status are presented in Table 1. No respondents rated their oral health status as bad, or very bad, and those categories were collapsed. More than half (53.1%) of the group rated their oral health status as excellent or very good. Almost 10% of the group could not rate their oral health status.

**Table 1:** Descriptive statistics for outcome variables (*N* = 710 participants)

			Gender		Statistical test / significance level	Total
			M	F		
Age (months)		Mean +/− SD	113.81 +/− 11.4	114.24 +/− 11.3	^*^ / 0.615	114.01 +/− 11.3
Perception of oral health	Excellent	% (*n*)	27.5% (103)	32.2% (108)	^**^ / 0.521	29.7 (211)
	Very good	% (*n*)	25.6% (96)	20.9% (70)		23.4 (166)
	good	% (*n*)	19.5% (73)	18.5% (62)		19.0 (135)
	average	% (*n*)	17.1% (64)	19.7% (66)		18.3 (130)
	I do not know	% (*n*)	10.4% (39)	8.7% (29)		9.6 (68)
BMI categories	normal weight	% (*n*)	76.3% (286)	78.2% (262)	^**^ / 0.293	77.2 (548)
	overweight	% (*n*)	14.4% (54)	19.1% (64)		16.6 (118)
	obese	% (*n*)	9.3% (35)	2.7% (9)		6.2 (44)
BMI Z-score		Mean +/− SD	0.97 +/− 1.38	0.78 +/− 1.34	^*^ / 0.068	0.88 +/− 1.36
dmft score		Mean +/− SD	1.71 +/− 1.97	2.08 +/− 2.13	^*^ / 0.017	1.89 +/− 2.06
DMFT score		Mean +/− SD	0.85 +/− 1.09	0.88 +/− 1.11	^*^ / 0.720	0.86 +/− 1.10
DAI score		Mean +/− SD	23.70 +/− 7.74	23.26 +/− 6.48	^*^ / 0.406	23.49 +/− 7.17
Orthodontic treatment need		% (*n*)	36.3 (136)	36.7 (123)	^**^ / 0.901	36.5 (259)
Total no of untreated decayed teeth		Mean +/− SD	1.74 +/− 1.83	2.14 +/− 2.09	^*^ / 0.006	1.93 +/− 1.97

Note: ^*^ t-test ^**^ Mann-Whitney

*SD* standard deviation

No significant differences were found between “I do not know” responders and lower class self-raters (collapsed good and average raters) (*p* > 0.05), therefore in further analysis for the prediction of lower class of self-perception of oral health, they were considered together with lower self-raters.

### Oral health

For the sample, dmft and DMFT scores and Dental Aesthetic Index (DAI) score were calculated and descriptive statistics are presented in Table 1.

Using the general linear model with gender (male vs female) and age groups (8–9 years vs 10–11 years) as factors, four different models were constructed for dmft score, DMFT score and DAI score and the total number of untreated decayed teeth. Only models constructed for dmft score (*p* = 0.017), DMFT score (*p* = 0.005) and for the total number of decayed teeth (*p* = 0.036) reached statistical significance (Table 2).

**Table 2:** General linear models (GLM) for oral health indicators as dependent variables by gender and age group (*N* = 710 participants)

	Variables	Categories		Mean +/− SD	Significance	Partial Eta Squared
dmft	Gender	male (375)		1.71 +/− 1.971	0.016	0.008
		female (335)		2.08 +/− 2.132		
	Age groups	8–9 years (449)		2.01 +/− 2.124	0.035	0.006
		10–11 years (261)		1.68 +/− 1.916		
	Gender ^*^ Age Group (Interaction)	male	8–9 years (242)	1.84 +/− 2.087	0.877	0.000
			10–11 years (133)	1.48 +/− 1.722		
		female	8–9 years (207)	2.20 +/− 2.156		
			10–11 years (128)	1.89 +/− 2.086		
DMFT	Gender	male (375)		0.85 +/− 1.095	0.587	0.000
		female (335)		0.88 +/− 1.105		
	Age groups	8–9 years (449)		0.76 +/− 0.960	0.001	0.017
		10–11 years (261)		1.05 +/− 1.285		
	Gender ^*^ Age Group (Interaction)	male	8–9 years (242)	0.78 +/− 1.010	0.272	0.002
			10–11 years (133)	0.98 +/− 1.228		
		female	8–9 years (207)	0.73 +/− 0.900		
			10–11 years (128)	1.12 +/− 1.344		
DAI	Gender	male (375)		23.70 +/− 7.744	0.328	0.001
		female (335)		23.26 +/− 6.478		
	Age groups	8–9 years (449)		23.97 +/− 7.550	0.021	0.008
		10–11 years (261)		22.68 +/− 6.406		
	Gender ^*^ Age Group (Interaction)	male	8–9 years (242)	23.98 +/− 8.015	0.349	0.001
			10–11 years (133)	23.21 +/− 7.227		
		female	8–9 years (207)	23.95 +/− 6.986		
			10–11 years (128)	22.14 +/− 5.397		
Decayed teeth	Gender	male (375)		1.74 +/− 1.835	0.007	0.010
		female (335)		2.14 +/− 2.091		
	Age groups	8–9 years (449)		1.98 +/− 1.944	0.306	0.001
		10–11 years (261)		1.84 +/− 2.011		
	Gender ^*^ Age Group (Interaction)	male	8–9 years (242)	1.79 +/− 1.849	0.972	0.000
			10–11 years (133)	1.63 +/− 1.811		
		female	8–9 years (207)	2.20 +/− 2.032		
			10–11 years (128)	2.05 +/− 2.188		

GLM dependent variable dmft, F(3) = 3431, *p* = 0.017, partial eta squared = 0.014, adjusted R squared = 0.010

GLM dependent variable DMFT, F(3) = 4337, *p* = 0.005, partial eta squared = 0.018, adjusted R squared = 0.014

GLM dependent variable DAI, F(3) = 2257, *p* = 0.080, partial eta squared = 0.010, adjusted R squared = 0.005

GLM dependent variable Decayed teeth, F(3) = 2858, *p* = 0.036, partial eta squared = 0.012, adjusted R squared = 0.008

For dmft score, significant differences were observed between genders, females having a higher dmft score, compared to males (*p* = 0.016, partial eta squared = 0.008) with the 8–9 year old age group having a significantly higher score than the 10–11 year old age group (*p* = 0.035, partial eta squared = 0.006), but their interaction was not statistically significant, *p* = 0.877. For DMFT score, significant differences were found between age groups, with the 10–11 year old group having a higher DMFT score, compared to 8–9 year old group (*p* = 0.001, partial eta squared = 0.017) but not between genders (*p* = 0.587), or the interaction between gender and age group (*p* = 0.272).

For the total number of untreated decayed teeth, significant differences were only observed between genders, females having a higher number, compared to males (*p* = 0.007, partial eta squared = 0.010) but the differences between age groups (*p* = 0.306) and their interaction (*p* = 0.877) were not statistically significant.

The general linear model for DAI score did not reach significance, *p* = 0.080 (Table 2).

### BMI status

Prevalence of overweight is 14.4% (54) for boys and 19.1% (64) for girls and the prevalence of overweight and obesity is 23.7% (89) for boys and (73) 21.8% for girls. For this sample, no statistical differences were found between genders related to BMI z-score (*p* = 0.068), nor between the proportion of BMI categories, between genders (*p* = 0.293) (Table 1).

### Body image dissatisfaction

Of all the students included in our study, 43.8% (311) are satisfied with their body image. 45.6% (324) reported they would like to be thinner (35.2% by 1 level, 8.6% by 2 levels and 1.8% by 3 levels) and 35.2% (250) that they wanted to be heavier (10.1% by 1 level and 0.4% by 2 levels).

General linear model was applied in order to quantify the influence of gender, BMI categories or their interaction on satisfaction levels. Gender (*p* = 0.549) and the interaction of gender and BMI categories (*p* = 0.578) did not contribute significantly to the model (Table 3).

**Table 3:** General linear model (GLM) for dependent variable body dissatisfaction level by gender, BMI categories and interaction of gender and BMI categories (*N* = 710 participants)

Variables	Categories		Mean body dissatisfaction level +/− SD	Significance	Partial Eta Squared
Gender	male (375)		0.49 +/− 0.889	0.548	0.001
	female (335)		0.44 +/− 0.852		
BMI categories	normal weight (548)		0.39 +/− 0.853	< 0.001	0.031
	overweight (118)		0.68 +/− 0.856^*^		
	obese (44)		0.93 +/− 0.925^*^		
Gender^*^ BMI categories	male	normal weight (286)	0.42 +/− 0.870	0.578	0.002
		overweight (54)	0.65 +/− 0.894		
		obese (35)	0.89 +/− 0.932		
	female	normal weight (262)	0.35 +/− 0.835		
		overweight (64)	0.70 +/− 0.830		
		obese (9)	1.11 +/− 0.928		

Note: *GLM* dependent variable Body dissatisfaction levels, F(5) = 5.213, *p* < 0.001, partial eta squared = 0.036

^*^mean body dissatisfaction significantly higher in overweight (*p* = 0.003) and obese (*p* = 0.001) compared with normal weight children, results adjusted by Sidak method for multiple comparisons

BMI categories significantly influenced the outcome, F(2) = 11.37, *p* < 0.001. The result was further explored with post-hoc tests with Sidak correction for multiple comparisons. Normal weight children had a significant higher level of satisfaction when compared with overweight children (mean difference = 0.29, *p* = 0.003) and with obese children (mean difference = 0.613, *p* = 0.001). The differences between levels of satisfaction in overweight and obese children were not statistically significant (mean difference = 0.323, *p* = 0.201).

For the body image dissatisfaction measure, the absolute discrepancy was also calculated to remove the direction and the extent of dissatisfaction.

### Predictors of poor self-perception of oral health status

In univariate unadjusted logistic regression, gender, BMI categories, DMFT and DAI score did not significantly contribute to the prediction of poor self-rated oral health. Untreated decayed teeth, dmft score and body dissatisfaction levels had significant contributions in univariate analysis (Table 4).

**Table 4:** Logistic regression results for the predictors of poor self-evaluation of oral health status (*N* = 710 participants)

	Unadjusted OR^*^	95% C.I. for OR	Model OR^**^	95% C.I. for OR
Gender (Male)	1.003	(0.746–1.347)	1.055	(0.776–1.435)
BMI categories	*p* = 0.745			*p* = 0.969
Overweight vs Normal weight	1.045	(0.702–1.557)	1.029	(0.682–1.552)
Obese vs Normal weight	1.268	(0.685–2.345)	1.076	(0.567–2.040)
dmft score	1.075	(1.000- 1.156)	.911	(0.806–1.030)
DMFT score	1.083	(0.947- 1.238)	.899	(0.763–1.058)
DAI	1.006	(0.985- 1.027)	1.004	(0.983–1.025)
Untreated decayed teeth	1.154	(1.068- 1.246)	1.293	(1.120–1.492)
Body image dissatisfaction	*p* = 0.006			*p* = 0.003
1 level vs no dissatisfaction	1.585	(1.157- 2.172)	1.605	(1.164–2.211)
2 levels vs no dissatisfaction	1.820	(1.059- 3.129)	2.040	(1.168–3.564)
3 levels vs no dissatisfaction	3.393	(1.023- 11.260)	3.617	(1.057–12.373)

Dependent variable: poor self-evaluation of oral health

Independent variables: Gender, BMI categories, dmft score, DMFT score, DAI score, total number of decayed teeth

^*^Unadjusted OR were calculated separately for each individual variable

^**^Model adjusted for gender, BMI categories, dmft score, DMFT score, DAI score; log likelihood chi-square: 31.364, Prob > chi-square: 0.001, Pseudo R-square: 0.043

In the model, when controlling for gender, BMI categories, dmft score, DMFT score and DAI score, the unique contributors were the number of untreated decayed teeth and the levels of dissatisfaction with the body (Table 4).

Body image dissatisfaction has a unique contribution in the prediction of poor self-rated health: the higher the level of dissatisfaction, the higher probability of a poor evaluation class: when compared with the students who did not exhibit dissatisfaction with their body image, those who had one level of dissatisfaction had an OR = 1.61, those with 2 levels of dissatisfaction had an OR = 2.04 and those with 3 levels of dissatisfaction had an OR = 3.61. Body image dissatisfaction had similar OR and 95% confidence intervals in adjusted and unadjusted models, showing that the relation is not confounded.

In the adjusted model, when controlling for dmf and DMF scores, for each extra untreated decayed tooth there is a 29% higher probability of poor self-rated oral evaluation. In the unadjusted model, when dmft and DMFT scores were not controlled for, the probability was lower, for each extra untreated decayed tooth there was a 15% higher probability of poor evaluation. In the correlation matrix, the mean total number of decayed teeth was correlated with the dmft score (tau = 0.688, *p* < 0.001) and the DMFT score (tau = 0.408, *p* < 0.001), therefore dmft score and DMFT score are confounders and have to be controlled for, in the prediction of poor self-rated oral health.

## Discussion

According to the literature [17], from the end of the first period of mixed dentition and the beginning of the second period of mixed dentition children with mixed dentition present an optimal morpho-functional and aesthetic balance, dento-alveolar harmony, balanced occlusal relations in the three planes, the dental upper arch circumscribing the dental lower arch with a vestibular cusp, the upper incisors covering the lower one with a contact point or incisal step of 1–2 mm, permanent molars in the cusp-groove or cusp-cusp relationship, fraenums labiis on the maxillary midline and skull base, and absence of diastema. The caries of the deciduous teeth disrupt the development of the dento-maxillary apparatus, this balance being lost.

For this age group, as far as it is known, self-rated oral health status has never been evaluated regarding its oral clinical indicators (carious experience and malocclusion) and body shape and body image dissatisfaction as contributing risk factors.

In this study, self-perception of oral health was not related to clinical epidemiological oral health indicators or dento-occlusal aesthetic indicators, with the exception of untreated decayed teeth. Although dmft Index, DMFT Index, dmft/DMFT Index have been used intensively in clinical settings in order to assess dental caries prevalence, as well as dental treatment needs among populations [18], for this age group, only the cumulated “decayed” component of both scores is associated with self-perceived oral health with an OR = 1.293, 95%CI (1.120, 1.492), as has been previously reported [19]. Since the proximal consequence of dental decay is pain [20], it is likely that the contribution of decayed untreated teeth to self-reported oral health is viewed by children through their subjective measure. Untreated cavitated dentine lesions and their consequences negatively influence the quality of life in children [21].

The presence of malocclusion did not relate to self-perception of oral health, although, in older adolescents and adults, malocclusion has physical, social, and psychological effects on oral conditions as well as on the quality of life [22]. It might be that at this age self-consciousness regarding facial aesthetics is not well established. The need for orthodontic treatment for this mixed dentition population is high (36,5%), possibly related to the fact that, orthodontic treatment is rarely applied in deciduous dentition and is more often recommended to be applied at phase I in early mixed dentition, as soon as the upper lateral incisors are erupted [23], so it is likely that our population is not well informed about orthodontic treatment. A recent governmental report [24] showed that only 10% of children with malocclusion and dental malposition benefited of orthodontic treatment in Romania. Children and adolescents mainly seek orthodontic treatment due to dissatisfaction with their dento-facial appearance, orthodontic counselling, and the influence of peers who wear braces [22].

Dissatisfaction with the body image is a complex indicator. During pre-pubertal and pubertal years, children’s bodies are in transition regarding shape, weight status and appearance. Children have a unique vision of reality. Body image is influenced by media, parents, peers, romantic peers, all of whom shape beliefs about the ideal body. Internalization and perceived pressure to align oneself to social norms can be used to explain the links between measured and perceived body weight [25, 26]. In this study, dissatisfaction with body image was associated with poor self-perception of oral health status. This relation can be explained through psychological traits such as self-esteem, life satisfaction, quality of life, sense of coherence, anxiety and depression [27–33]. Several Delphi consensus studies have agreed that the prevention of the onset of body dissatisfaction [34] and childhood depression and anxiety disorders [35] can be achieved through a variety of positive parenting strategies that include healthy eating patterns discussions, establishing and maintaining a good relationship, being involved and supporting increasing autonomy, establishing family rules and consequences, and encouraging habits of good health.

Our study had several limitations. First, it is a cross sectional study. Then, a selection bias has to be considered, since our participants were self-selected from pupils attending primary school. Since our results are based on participants from urban area, which have higher access to healthcare services, it will be important to have a broader image of the impact of a diversified environment. The lack of data on parents’ education level, on indicators of socio-economic status, socio-cultural status, oral health-related behaviours, are other limitations. However, the analysis was adjusted by gender, weight categories and oral clinical indicators.

## Conclusions

Oral health perception is a good and cheap indicator of the tooth decay treatment need in pre-adolescents with mixed dentition, which can impact oral health outcomes and reduces risks of morbidity. Oral health surveillance in mixed dentition population should include information on self-perceived oral health. The association of self-perception of oral health with dissatisfaction with body image, but not BMI, underlines that even at this age, oral health is included in the general perception of body status. Given that body image dissatisfaction may exacerbate emotional distress, and because in adolescence there is an increased risk of developing a negative body image and an increased vulnerability to social-cultural influences, this should be a primary goal for clinical interventions. Planning for medical intervention should include therapeutic interventions appropriate to needs and motivational particularities of preadolescents.

## Abbreviations

BMI: Body mass index
CI: Confidence interval
d/D: Decayed teeth (for deciduous dentition in lower case and for permanent teeth in uppercase)
DAI: Dental aesthetic index
dmft/DMFT: Decayed, missing, filled teeth (for deciduous dentition in lower case and for permanent teeth in uppercase)
f/F: filled teeth (for deciduous dentition in lower case and for permanent teeth in uppercase)
GLM: General linear model
m/M: missing teeth (for deciduous dentition in lower case and for permanent teeth in uppercase)
OR: Odds ratio
SD: Standard deviation
WHO: World Health Organization
